# The Role of Coronary Imaging in Chronic Total Occlusions: Applications and Future Possibilities

**DOI:** 10.3390/jcdd11090295

**Published:** 2024-09-21

**Authors:** Giuseppe Panuccio, Youssef S. Abdelwahed, Nicole Carabetta, Ulf Landmesser, Salvatore De Rosa, Daniele Torella

**Affiliations:** 1 Department of Experimental and Clinical Medicine, Magna Graecia University, 88100 Catanzaro, Italy; dtorella@unicz.it; 2 Department of Cardiology, Angiology and Intensive Care Medicine, Deutsches Herzzentrum der Charité, 12200 Berlin, Germany; youssef.abdelwahed@dhzc-charite.de (Y.S.A.); ulf.landmesser@dhzc-charite.de (U.L.); 3 Department of Medical and Surgical Sciences, Magna Graecia University, 88100 Catanzaro, Italy; nicole.carabetta95@gmail.com (N.C.); saderosa@unicz.it (S.D.R.)

**Keywords:** chronic total occlusions, CTOs, coronary imaging, IVUS, OCT, computed tomography, precision medicine

## Abstract

Chronic total occlusions (CTOs) represent a challenging scenario in coronary artery disease (CAD). The prevalence of CTOS in patients undergoing coronary angiography underscores the need for effective diagnostic and therapeutic strategies. Coronary angiography, while essential, offers limited insights into lesion morphology, vessel course, and myocardial viability. In contrast, coronary imaging techniques—including optical coherence tomography (OCT), intravascular ultrasound (IVUS), and coronary computed tomography angiography (CCTA)—provide comprehensive insights for each stage of CTO percutaneous coronary intervention (PCI). OCT facilitates the assessment of plaque morphology and stent optimization, despite low evidence and several limitations in CTO-PCI. IVUS offers deeper penetration, allowing managing proximal cap scenarios and guiding subintimal navigation. CCTA provides a non-invasive, three-dimensional view of coronary anatomy, enabling the precise evaluation of myocardial mass at risk and detailed procedural planning. Despite their individual limitations, these imaging modalities have enhanced the success rates of CTO-PCI, thus reducing procedural and long-term complications and improving patient outcomes. The future of CTO management lies in further technological advancements, including hybrid imaging, artificial intelligence (AI) integration, and improved fusion imaging. These innovations promise to refine procedural precision and personalize interventions, ultimately improving the care of patients with complex coronary artery disease.

## 1. Introduction

Chronic total occlusions (CTOs) represent a challenging scenario in coronary artery disease (CAD), characterized by increasing morbidity and adverse clinical outcomes [1]. The prevalence of CTOs among patients undergoing coronary angiography is about 16–18%, highlighting the importance of diagnostic and therapeutic strategies in this setting [2]. Coronary revascularization, including coronary artery bypass grafting (CABG) and percutaneous coronary intervention (PCI) represent a cornerstone treatment in CAD, including CTOs [3,4,5]. The clinical implications of CTOs are mainly a significant increase in angina symptoms and exercise tolerance, thus reducing quality of life [6]. However, despite the lack of randomized data, observational studies have shown a significant improvement in ejection fraction and in long-term survival in patients undergoing the successful recanalization of a CTO [7,8,9]. Coronary angiography has been the gold standard for the anatomical assessment of CTOs for years. However, it provides limited information about lesion’s morphology, vessel course and myocardial viability, as well as about stent implantation and optimization. This limitation has been overcome by coronary imaging techniques, which provide useful information in every step of CTO-PCI, ranging from pre-procedural planning to enabling successful CTO crossing and achieving procedural success. Since its complexity, the management of CTOs often benefits from the support of coronary imaging in several phases, even before the CTO-PCI procedure. Non-invasive imaging modalities such as coronary computed tomography angiography (CCTA) offer comprehensive insights into CTO vessel and myocardial perfusion [10]. Invasive imaging techniques, including intravascular ultrasound (IVUS) and optical coherence tomography (OCT) provide detailed information of plaque composition, vessel size, and stent optimization. Moreover, IVUS can guide CTO crossing by extra-plaque techniques [11]. Since its many applications in CTO-PCI, coronary imaging has become an essential tool in this clinical scenario, especially in complex CTO lesions. Therefore, the aim of this work is to explore the current applications of coronary imaging techniques in the context of CTO-PCI, highlighting the strengths and limitations of each modality. Furthermore, we provide a discussion about emerging technologies related to coronary imaging and future perspective that may shape the field of CTO intervention.

### Optical Coherence Tomography

**Figure 1:**
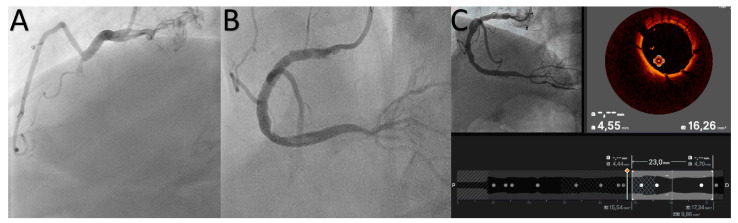
CTO-PCI of a right coronary artery (**A**,**B**) and OCT showing a good stent struts apposition and stent area (**C**).

OCT is a high-resolution intravascular imaging modality which uses near-infrared light to produce cross-sectional images of the coronary artery. Thanks to its high resolution (10–20 μm), OCT enables a detailed assessment of arterial wall and plaque morphology [12,13], as well as the optimization of stent deployment [8]. Although the evidence in the context of CTO-PCI is limited in respect to the other coronary imaging modalities, OCT has several applications. During CTO-PCI, the guidewire crossing of the lesions is a major challenge. OCT can help to confirm the intraluminal position of the guidewire, thereby decreasing the risk of subintimal course and potential perforation or dissection [14]. Moreover, in extra-plaque techniques used to cross the CTO lesion, case reports showed the feasibility of an antegrade dissection-and re-entry (ADR) approach through a novel OCT-guided CTO re-entry device, which provides real-time image guidance for true lumen re-entry [15]. After successful guidewire crossing, OCT can help to analyze the CTO-plaque, assessing its composition (fibrous, calcific, lipid-rich). In some CTOs, thanks to its high-resolution, OCT can identify microchannels within the context of the plaque, which sometimes may facilitate the progression of soft, low-profile guidewires [16]. Finally, OCT favors stent deployment and optimization (Figure 1A–C), which resulted in a significantly lower risk of target vessel failure (TVF) in a prespecified sub-study of the RENOVATE-COMPLEX-PCI trial [17]. However, despite its applications, OCT presents several limitations in CTO-PCI. First, its limited penetration depth (1–2 mm) could be a drawback in cases with large or heavily calcified plaques where a deeper visualization is required. Second, OCT imaging requires the clearance of blood from the vessel, typically achieved through contrast injection. This requirement can increase the risk of extending a dissection rim when it has been caused by guidewire crossing. Further studies will provide further data about OCT use in CTO-PCI.

## 2. Intravascular Ultrasound

**Figure 2:**
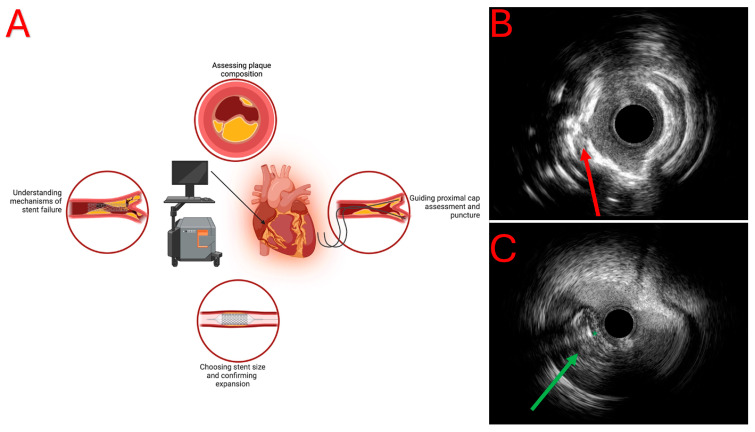
(**A**) IVUS applications in CTO-PCI. (**B**) Practical IVUS examples a heavily calcified CTO-plaque with evidence of calcium fracture after rotational atherectomy (red arrow). (**C**) Tip detection antegrade dissection and re-entry (TD-ADR) showing an IVUS catheter in the subintimal space guiding the penetration of the wire (green mark) in the true lumen (green arrow).

IVUS is an imaging modality that uses high-frequency sound waves to create detailed cross-sectional images of the coronary arteries. IVUS catheters are equipped with ultrasound transducers that emit sound waves, which reflect off the vessel wall and the surrounding structures, and the reflected signals are then converted into images that provide the real-time visualization of the vessel lumen and wall [18,19]. IVUS catheters can be either phased array or mechanical. Phased array catheters present multiple transducers that electronically steer the ultrasound beam, while mechanical catheters have a single rotating transducer [20]. Thanks to its high penetration, IVUS has several applications in CTO-PCI (Figure 2A,B).

### 2.1. Proximal Cap Ambiguity

The proximal cap ambiguity is defined as the inability to clearly define the proximal entry point of the plaque, with a prevalence in approximately 30% of CTOs [21]. The presence of proximal cap ambiguity is also associated with higher rates of major adverse cardiac events (MACE) and retrograde crossing attempts [21]. In this scenario, an IVUS catheter can be advanced over a guidewire into a side branch adjacent to the proximal cap. Accordingly, subsequent IVUS pullback allows us to correctly assess the proximal cap and its composition, therefore, helps in making the choice between a high-penetration guidewire or a soft, polymer-jacketed guidewire [22].

### 2.2. Support in Antegrade Dissection and Re-Entry (ADR)

During CTO-PCI, the operators may direct the guidewire to the subintimal space in order to enable re-entry into the intimal space through different ways: (a) a dedicated device such as the Stingray™ (Boston Scientific, Marlborough, MA, USA) balloon or a dual lumen microcatheter; (b) redirecting the guidewire; (c) using another guidewire (parallel wire technique); or (d) using IVUS guidance in order to re-enter the intimal space with another guidewire. This last approach, called tip detection antegrade dissection and re-entry (TD-ADR) represents one of the most recent techniques in the CTO-PCI field and requires IVUS guidance to guide a stiff guidewire to re-enter the intimal space (Figure 2C) [23,24]; this approach shows higher procedural success rates and significantly lower procedural time in comparison to the conventional Stingray-assisted ADR [25].

### 2.3. Support in Reverse Controlled Antegrade and Retrograde Tracking (CART)

The retrograde approach allows us to cross the CTO distally through the collaterals, which could be septal, epicardial, or bypass collaterals. Thanks to this approach, the success rates of CTO-PCI significantly increased over years [26]. The indications for the use of the retrograde approach range from proximal cap ambiguity, long CTO lesions, bifurcation at the distal cap, and aorto-ostial lesions [27]. During this approach, the retrograde guidewire can be used: (a) as a marker for directing an antegrade wire which crosses the lesion; (b) for a retrograde true lumen puncture of the distal cap; or (c) for a retrograde dissection and re-entry approach, with the reverse CART being the most common strategy used in this setting. This consists of an antegrade knuckled guidewire delivered in the subintimal space, with a balloon sized 1:1 deflated on the antegrade wire which creates a connection between the subintimal and the intimal space. Subsequently, the retrograde wire advances from the distal to the proximal true lumen through the created space [28]. In this scenario, IVUS can help to define the position of both antegrade and retrograde guidewires and to identify plaque morphology and vessel size. This last phase is crucial for reversing the CART technique, because IVUS helps to identify the best segment of the vessel where a space connection between subintimal and intimal space can be created and can guide the selection of the appropriate guidewire to perform CTO crossing [29].

### 2.4. Stent Deployment and Optimization

After CTO crossing, IVUS allows the precise measurement of a vessel’s dimension as well as the lesion’s length to precisely identify stent’s size and its landing zones. Since the geographical miss, stent under-sizing or malposition are the major predictors of stent thrombosis, IVUS acquires a significant impact in this scenario [30,31,32]. Although IVUS guidance in complex CAD is recommended since it significantly improves clinical outcomes, there is limited evidence regarding the clinical impact of IVUS in CTO-PCI [33]. Table 1 shows the studies reporting a comparison between IVUS- and angiography-guided CTO-PCI and shows a significant reduction in major adverse cardiac events (MACE), myocardial infarction, stent thrombosis, and in-stent late luminal loss [34,35,36,37,38]. Thanks to the appropriate vessel size and length estimation, summoning the evidence of recent trials showed that an IVUS-guided CTO-PCI is related to higher stent length and stent diameter, resulting in evidence of a significantly lower risk of stent thrombosis [39].

### 2.5. IVUS Limitations

Despite its several applications, IVUS has some limitations that must be considered, including lower resolution compared to OCT, which may preclude the detailed visualization of microstructures within the plaque. IVUS also requires significant operator expertise for accurate image interpretation, especially in subintimal approaches, leading to a variability in outcomes.

## 3. Coronary Computed Tomography Angiography (CCTA)

**Figure 3:**
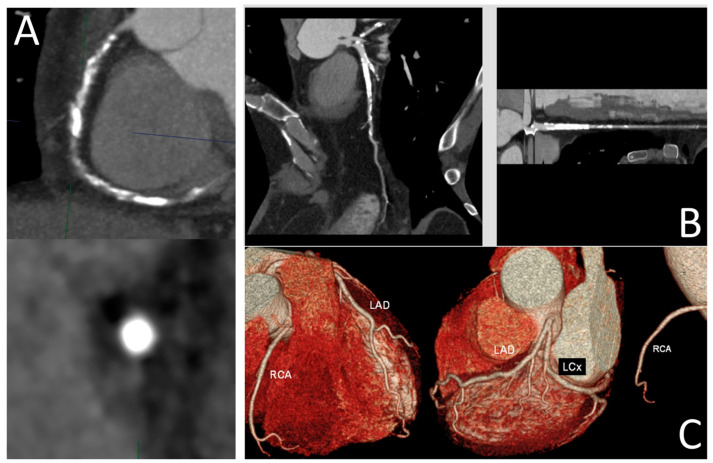
(**A**) visualization of a high-calcified CTO plaque of a right coronary artery with the slab maximum intensity projection (MIP) technique; (**B**) curved and stretched multi-planar reconstruction of a LAD-CTO; (**C**) three-dimensional reconstruction of coronary arteries through the volume rendering approach.

CCTA is a non-invasive imaging modality which provides detailed three-dimensional images of the coronary arteries [40]. Utilizing CT technology, CCTA acquires high-resolution images that can be reconstructed to evaluate coronary anatomy, plaque characteristics and vessel course [41]. The extracted CCTA images of the coronary arteries are paired utilizing bifurcation points as markers to fluoroscopic diastolic images in order to balance breathing and cardiac rhythm. Co-registration between CCTA and fluoroscopic images facilitates the detection of angiographic projections that reduce vascular overlap and the foreshortening of the segment of interest. Through automatic softwares, it is feasible to reconstruct the centerline in the non-occluded segments of the vessel by identifying the contrast-filled lumen, while in the occluded segments, the difference in the Hounsfield Unit (HU) related to calcifications can aid in recognizing the vessel lumen of the CTO [42,43]. Thanks to its visualization modalities, CCTA allows for the multi-parametric evaluation of the CTO lesion. Slab maximum intensity projection (MIP) generates a projection image of a slab of tissue, highlighting the most intense structures. This method is effective in identifying calcified plaques and visualizing the overall vessel architecture [44] (Figure 3A). Curved multiplanar reconstruction (MPR) allows for the visualization of the coronary arteries along their natural course, providing a continuous view of the vessel, while stretched MPR visualizes the artery in a straightened format, making it easier to measure lesion length and identifying the occlusion site (Figure 3B). Finally, volume rendering creates a three-dimensional representation of the coronary arteries by combining data from multiple CT slices. This provides a comprehensive view of the vessel anatomy (Figure 3C). By providing a three-dimensional rendering of the coronary arteries, CCTA allows a precise identification of the occluding segments and their supply territories, enabling the quantification of the amount of myocardium at risk.

CCTA can be used to analyze the characteristics of the CTO segment before PCI. The non-invasive estimation of the proximal cap location and its features, the precise artery trajectory, and the calcium distribution are made possible by CCTA [45]. In fact, with traditional angiography, these data are less accurate. Thus, CCTA provides a better assessment of complex CTO, which may lead to shorter procedural time, less radiation and contrast injection, and consequently, the better planning of the procedure [46,47]. A functional assessment of wall motion and myocardial perfusion is also made possible by the ongoing advancement of scanner technologies [48]. Thanks to CCTA, several predictors of procedure’s complexity have been identified, including the extent of calcification, lesion length, negative remodeling, blunt stump, vessel bending and the presence of side branches [49,50,51]. Among these risk factors of procedural complexity, calcification has several implications. Heavy calcifications within the CTO-plaque may impede device delivery, often forcing operators to adopt extra plaque approaches, burdened by higher periprocedural risks and worse outcomes [52]. Severe calcifications can compromise optimal stent expansion, which is a major risk factor for stent thrombosis [30]. CCTA allows us to adequately identify calcium burden, allowing an adequate planning of the procedure. Particularly, CCTA allows us to identify heavy calcification with a circular disposition of 360° and on the 100% of cross-sectional area (CSA), defined as “full-moon” calcifications, which have been shown to be independent predictors of an inability to cross the lesion or the need for intense debulking devices such as intravascular lithotripsy and rotational atherectomy, thus increasing the risk of procedural complications like coronary perforations [53,54]. In the evaluation of CTOs, several scores are useful tools to assess a lesion’s complexity. The most used score is the J-CTO score, an angiographic score that identifies the probability of successful guidewire crossing within 30 min [55]. Recently, two CCTA-based scores (CT-RECTOR and KCCT) have been developed and have demonstrated a better predictive performance than the J-CTO angiographic score [56,57]. CT-RECTOR score predicts 30 min wire crossing by examining anatomical features such as stump morphology, multiple occlusions, the calcification extent of >50% of the vessel area, bending within the occlusion of greater than 45° and clinical characteristics as prior attempt, and the duration of CTO of >12 months [58]. In addition, the KCCT score considers other two features as the presence of an adjacent side branch and gives a supplementary point to central calcifications (360° and 100% CSA; Table 2). Recent evidence has examined the success rate of CTO-PCI after a pre-procedural CCTA, with higher success rates and fewer complications in the CCTA group compared to the angiography-only group [59]. According to these mentioned data, it seems feasible to apply CCTA in CTO-PCI procedures, especially for patients with high-score CTOs (e.g., J-CTO ≥ 2), in cases of prior failed procedures, in patients with previous CABG, and in situations with poor visualization of the vessel’s course [60]. Further studies are ongoing (NCT05364827); these will examine patients randomly assigned to CCTA-guided CTO-PCI or only PCI. The primary endpoint will be the success rate, while the secondary endpoints will be the assessment of angina valued by the Seattle Angina Questionnaire, the need for a second CTO procedure, procedural complications, wire crossing time, and procedural time. The main limitation of the use of CCTA in this field is the additional use of radiation and contrast agents, although modern CT scanners have significantly decreased radiation and contrast while simultaneously enhancing picture quality. Likewise, we should expect further improvements in the fusion imaging of CCTA and fluoroscopy, and future research should identify the features of lesions that will benefit most from these new techniques.

## 4. Discussion

Coronary imaging techniques have revolutionized approaches to managing CTOs in CAD. Each modality offers unique advantages and plays a crucial role in different phases of CTO-PCI (Table 3). The importance of coronary imaging in this field has been widely recognized, not only for the increased technical success rates provided by these approaches. In fact, coronary imaging guidance has shown not only a significant improvement in quality of life [61,62] but also a significant reduction in procedural and long-term complications in respect to an angiography-only guidance, which is pivotal in the context of CTO-PCI. In more detail, in the recent OCCUPI trial, an OCT guidance led to a significant reduction in a composite endpoint of cardiac death, myocardial infarction, ischemia-driven target lesion revascularization, and stent thrombosis in complex CAD, including patients with CTOs [63]. Similarly, several studies showed a lower incidence of stent-thrombosis after IVUS guidance in CTO-PCI [39]. Finally, in patients undergoing CCTA before CTO-PCI, lower rates of intraprocedural complications like coronary perforations were observed [59]. Despite their individual strengths, these imaging modalities also present some limitations that should be carefully considered when planning and performing CTO-PCI. OCT, with its high-resolution, plays an important role in identifying microchannels and for stent optimization. However, its limited penetration depth restricts its utility in heavily calcified lesions and the requirement for blood clearance during imaging may increase procedural complications due to the enlargement of dissections. Therefore, while OCT plays a role in the later stages of CTO-PCI, its use during crossing phase remains limited. IVUS, with its deeper penetration, offers comprehensive insights into vessel’s lumen and wall, which is particularly useful in ambiguous proximal cap scenarios and subintimal navigation during ADR or retrograde approach. IVUS-guided CTO-PCIs have also shown to reduce the short- and long-term complications of stent implantation [39]. However, operator-dependent interpretations may cause variability in outcomes, which may be mitigated by future technological developments and artificial intelligence (AI) algorithms, and its low resolution may miss microchannels or small dissections. CCTA serves as a useful non-invasive tool, providing the three-dimensional reconstructions of coronary anatomy, plaque characteristics, and vessel direction. The possibility of identifying myocardial mass at risk, combined with advance imaging techniques such as slab MIP, curved and stretched MPR, and volume rendering, allows for detailed procedural planning and risk stratification. Despite these advancements, several challenges persist, including cost, accessibility, and operator expertise. The high costs of imaging devices and limited availability can restrict their routine use, particularly in resource-limited settings. Additionally, the learning curve associated with coronary imaging can result in variability in outcomes, especially in less experienced operators. Further training protocols and the standardization of imaging protocols will help to reduce these issues, enabling more consistent results across centers, and aiding in the dissemination of these fundamental tools to achieve procedural success and increase safety.

## 5. Future Possibilities

The future of coronary imaging in CTO intervention is poised for significant advancements, driven by technological innovation and interdisciplinary research. The integration of multiple imaging modalities with hybrid assessment holds promises to provide insights that leverage the strengths of each technique. Advancements in imaging technology, such as high-definition IVUS and ultra-high-resolution CCTA will offer more detailed images during CTO analyses. Further, AI and machine learning (ML) algorithms are set to reform the analysis and interpretation of coronary imaging, improving the reconstruction of the vessel, reducing postprocessing time and favoring the prediction of the successful percutaneous recanalization of CTO lesions [64,65,66]. Automated image segmentation and plaque characterization can reduce operator dependency and enhance diagnostic accuracy. The development of fusion imaging techniques that combine CCTA with real-time fluoroscopy can provide dynamic, real-time guidance during CTO-PCI. This approach can help reduce procedural time, minimize radiation exposure, and improve the accuracy of guidewire crossing and stent deployment. Continued innovation, research and collaboration across disciplines will be essential in unveiling the full potential of these advanced imaging techniques, ultimately improving the management of this complex scenario.

## 6. Conclusions

Advanced coronary imaging techniques have significantly improved the management of CTOs. OCT, IVUS, and CCTA provide irreplaceable support, from non-invasive anatomical evaluation and procedural planning detailed to plaque assessment and stent optimization. While each modality has its limitations, their integration into clinical practice has enhanced procedural success, safety, and patient outcomes. The development of AI and ML algorithms, along with fusion imaging approaches, will overcome the limitations of these techniques, broadening their use and significantly increasing operators’ expertise and confidence in performing CTO-PCI. Future advancement in these technologies promises greater precision and personalized approaches to CTO interventions.

## Figures and Tables

**Table 1:** Studies reporting a comparison between IVUS- and angiography-guided CTO-PCI.

Study	K-CTO	CTO-IVUS	Air-CTO	Progress-CTO	Kalogeropoulos et al. [38]
Year	2014	2015	2015	2020	2021
Study type	Observational	Randomized Controlled Trial	Randomized controlled trial	Observational	Observational
Sample Size	402 IG: 201 AG: 201	402 IG: 201 AG: 201	230 IG: 115 AG: 115	922 IG: 344 AG: 578	364 IG: 182 AG: 182
Follow Up (Years)	2	1	1	1	4
Primary Endpoint	Definite or probable stent thrombosis	Cardiac Death	in-stent late lumen loss (LLL)	CD, MI, TVR	All cause death, CD, MI, TVR
Procedural Success	**NR**	IG 99 AG 98	IG 91 AG 68	**NR**	NR
Retrograde Approach (%)	**NR**	IG: 7 AG 9.5	IG: 10.4 AG: 19.1	IG: 28.8 AG: 21.4	25.5 IG: 30.2 AG: 20.9
Anterograde Approach (%)	**NR**	IG: 93 AG: 90.5	IG: 89.6 AG: 80.9	IG: AWE 53.5 ADR:17.4 AG: AWE 57.1 ADR: 19.8	IG: AWE 60.4 ADR: 9.3 AG: AWE 69.2 ADR: 9.9
Second-Generation DES (%)	100	100	IG 27.8 AG 20.0	**NR**	100

CD: cardiac death; MI: myocardial infarction; TVR: target vessel revascularization; AWE: antegrade wire escalation; ADR: antegrade dissection re-entry; IG: intravascular ultrasound-guided group; AG: angiography-guided group.

**Table 2:** Overview of the CT-based CTO scores, along with the angiography-based J-CTO score.

	J-CTO Score	CT-RECTOR Score	KCCT Score
Morphology	☐ Tapered stump (0) ☐ Blunt stump (1)	☐ Blunt stump (1)	☐ Blunt stump (1)
Calcification	☐ Calcification within the CTO segment (1)	☐ Calcification extent>50% of the vessel CSA (1)	☐ Calcification with encircling <180° or CSA<50% (0) ☐ Peripheral calcification (≥180° or CSA≥50%) (1) ☐ Central calcification (360° and CSA 100%) (2)
Bending	☐ Bending ≥45 degrees (1)	☐ Bending ≥45 degrees (1)	☐ Bending > 45 degrees (1)
Length	☐ <20 mm (0) ☐ ≥20 mm (1)		☐ <15 mm (0) ☐ ≥15 mm (1)
Proximal Adjacent Side Branch			☐ No (0) ☐ Yes (1)
Re-Attempt	☐ Re-try lesion (1)	☐ Re-try lesion (1)	☐ Re-try lesion (1)
Occlusion		☐ Multiple occlusions (1)	
Duration		☐ Duration of CTO ≥12 months or unknown (1)	☐ Duration of CTO ≥12 months or unknown (1)
Total Score	☐ 0 Easy ☐ 1 Intermediate ☐ 2 Difficult ☐ ≥3 Very Difficult	☐ 0 Easy ☐ 1 Intermediate ☐ 2 Difficult ☐ ≥3 Very Difficult	☐ 0 Easy ☐ 1 Intermediate ☐ 2 Difficult ☐ 3 Very Difficult ☐ ≥4 Extremely Difficult

**Table 3:** Basic principles, strengths and weaknesses of coronary imaging techniques in CTO-PCI.

Imaging Modality	Basic Principles	Strengths	Weaknesses
OCT (Optical coherence tomography)	Near-infrared light	-High-resolution (10-20 μm), allowing detailed assessment of plaque morphology -Detection of microchannels, thrombus, edge dissection -Useful for guiding stent deployment/optimization	-Limited penetration depth -Requires blood clearance with contrast, may enlarge dissections -Supplementary contrast use may impair renal function
IVUS (Intravascular ultrasound)	High-frequency sound waves	-Good penetration depth -Assessment of vessel size, plaque burden, calcification -Useful for guiding stent deployment/optimization -Helpful for solving proximal cap ambiguity -Support in retrograde and antegrade dissection and re-entry (ADR) approaches, including tip-detection ADR	-Lower resolution comparing to OCT -Interpretation is operator-dependent -Unable to detect thin fibrous caps or microcalcifications
CCTA (Coronary Computed Tomography Angiography)	X-rays	-Non-invasive modality -Slab maximum intensity projection (MIP) allows high-quality images. -Curved and stretched multi-planar (MPR) reconstructions allow easy measurement of occlusion length. -Volume rendering (VR) creates a three-dimensional view of the vessel. -Identification of calcium patterns around the CTO-lesion. -Simulation of angiographic views, friendly to interventional cardiologists. -CCTA based scores (KCCT and CT-rector) predict success rates.	-Lacking real-time imaging during PCI -Contrast and radiation exposure -Blooming effect due to heavy calcification may reduce diagnostic accuracy -Training needed to use CCTA programs -High costs

## Data Availability

No new data were created or analyzed in this study.
